# Comparison of [^99m^Tc]Tilmanocept and Filtered [^99m^Tc]Sulfur Colloid for Identification of SLNs in Breast Cancer Patients

**DOI:** 10.1245/s10434-014-3892-2

**Published:** 2014-07-29

**Authors:** Jennifer L. Baker, Minya Pu, Christopher A. Tokin, Carl K. Hoh, David R. Vera, Karen Messer, Anne M. Wallace

**Affiliations:** 1 Department of Surgery, University of California, San Diego, La Jolla, CA 92093 USA; 2 Division of Biostatistics and Bioinformatics, University of California, San Diego, La Jolla, CA USA; 3 Department of Radiology, University of California, San Diego, La Jolla, CA USA; 4 Molecular Imaging Program, University of California, San Diego, La Jolla, CA USA

## Abstract

**Background:**

The efficacy of sentinel lymph node (SLN) surgery requires targeted removal of first-draining nodes; however, frequently more nodes are removed than necessary. [^99m^Tc]tilmanocept (TcTM) is a molecular-targeted radiopharmaceutical specifically designed for SLN mapping. We evaluated technical outcomes of SLN biopsy in breast cancer patients mapped with TcTM + vital blue dye (VBD) versus filtered [^99m^Tc]sulfur colloid (fTcSC) + VBD.

**Methods:**

There were 84 versus 115 patients in the TcTM versus fTcSC cohorts, respectively. Main measures were the number of SLNs removed per patient and factors influencing number of nodes removed. We also evaluated whether the radiotracer injected affected the proportion of positive nodes removed in node-positive patients.

**Results:**

Fewer nodes were removed among patients mapped with TcTM compared to fTcSC (mean TcTM: 1.85 vs. fTcSC: 3.24, *p* < 0.001). Logistic regression analysis adjusted for tumor characteristics showed that injection of fTcSC (*p* < 0.001) independently predicted removal of greater than 3 nodes. A similar proportion of patients was identified as node-positive, whether mapped with TcTM or with fTcSC (TcTM: 24 % vs. fTcSC: 17 %, *p* = 0.3); however, TcTM detected a greater proportion of positive nodes among node-positive patients compared with fTcSC (0.73 vs. 0.43, *p* = 0.001).

**Conclusions:**

Patients undergoing SLN biopsy with TcTM required fewer SLNs to identify the same rate of node-positive patients compared with fTcSC in breast cancer patients with similar risk of axillary metastatic disease. These data suggest that a molecularly targeted mechanism of SLN identification may reduce the total number of nodes necessary for accurate axillary staging.

The efficacy of sentinel lymph node (SLN) biopsy to accurately assess the pathologic status of the axilla while removing minimal nodes depends on the ability of a mapping agent to identify only clinically relevant (i.e., first draining) nodes.^1^ Limitations with current standard mapping agents are related to their particulate nature and include persistent radioactivity at the site of injection interfering with precise SLN identification and radiolabeling of higher echelon nodes.^1^,^2^ These limitations contribute to lower accuracy rates and removal of unnecessary nodes.^3^–^7^

[^99m^Tc]tilmanocept (TcTM) is a receptor-targeted radiopharmaceutical that was designed to improve the specific targeting of SLNs. It is a small synthetic molecule (molecular diameter 7.1 nm) that accumulates in lymphatic tissue by binding mannose receptors (CD206) expressed on reticuloendothelial cells within lymph nodes.^8^ The small size allows for more rapid clearance from its injection site compared with radiolabeled sulfur colloid, and its specific binding to lymphatic tissue allows for sustained SLN uptake in first echelon nodes.^2^,^9^–^11^ TcTM received FDA approval in May 2013.

In this study, we compared the technical and pathologic outcomes among clinically node-negative breast cancer patients who underwent SLN biopsy with TcTM + vital blue dye (VBD) vs. filtered [^99m^Tc]sulfur colloid (fTcSC) + VBD at a single institution. We evaluated whether the choice of radiotracer affected the rate of identified node-positive patients, the number of excised SLNs, and the proportion of positive nodes removed in the node-positive patients.

## Materials and Methods

### Patients

All breast cancer patients who underwent SLN biopsy at UCSD with TcTM as part of two highly similar clinical trials (June 2008–June 2009; July 2010–April 2011) were identified from a prospectively maintained database.^12^ A comparison cohort was comprised of consecutive breast cancer patients undergoing SLN biopsy with TcSC during the 1-year period that succeeded conclusion of the later trial (March 2011–March 2012). The UCSD institutional review board approved a retrospective review of patient data for this study. Inclusion criteria included female patients with biopsy-proven invasive breast cancer who had SLN biopsy as part of their primary surgical procedure. Patients with known axillary lymph node metastasis before surgery, patients with T4 or inflammatory breast cancer, patients who received neoadjuvant chemotherapy, and patients with a history of prior breast or axillary surgery were excluded from analysis. Additionally, because all patients mapped with TcTM received intraoperative injection of VBD in addition to radiotracer injection as part of the clinical trial design, patients mapped exclusively with TcSC without VBD were eliminated from analysis.

### Procedures

Patients received 0.5 mCi of TcTM or 0.5 mCi of fTcSC by intradermal injection overlying the tumor or biopsy site. All SLN biopsy operations were performed within 1–12 h of radiopharmaceutical injection using standard technique by one of two surgeons, each of whom have more than 10 years of SLN biopsy experience. Before skin incision, patients underwent an intradermal injection of 2–4 ml of isosulfan blue dye (Lymphazurin, US Surgical Corporation, Norwalk, CT, USA) around the primary tumor or biopsy site. Sentinel nodes were defined as any blue or hot node. Hot nodes were considered any node greater than 50 counts per 2 s and greater than 10 % of the node with the highest count rate. Nodes that were hard or suspicious also were removed and deemed as SLN regardless of radioactivity or blue dye. The number of SLNs per patient was recorded according to the surgeon-determined count in the operating room. All removed lymph nodes were sent to pathology for hematoxylin and eosin staining and immunohistochemical analysis. A positive sentinel node was defined as any SLN that contained metastasis >0.2 mm.

Demographic and clinicopathologic characteristics obtained from retrospective UCSD chart review included year of diagnosis, age at diagnosis, race, body mass index (BMI), primary tumor size, tumor location, histologic subtype (ductal, lobular, mixed/tubular), tumor grade (modified-bloom Richardson grade), estrogen and progesterone receptor (ER/PR) and Her2/Neu (Her2) status, and presence of lymphovascular invasion (LVI). Lymph node characteristics included the number of SLN removed (surgeon count) and the number of positive SLN removed (metastasis >2 mm).

### Statistical Analysis

Patients were analyzed according to use of TcTM or fTcSC for node mapping. Chi square analysis or Fisher’s exact test was used to compare demographic and clinicopathologic factors between groups. Factors related to number of nodes (≤3 vs. >3) removed were examined using multivariate logistic regression. We included in the model all factors significant in univariate *χ* ^2^ tests at *p* < 0.1. Wilcoxon rank-sum tests were used to compare the two groups regarding the total number of nodes removed and the proportion of positive nodes removed among node-positive patients.

We further used a zero-inflated negative binomial (ZINB) model to compare the probability of a patient being node positive and also the fraction of positive nodes among those removed, for node-positive patients, after adjusting for other factors.^13^–^15^ A ZINB model incorporates two components: (1) logistic regression (the zero component of the model) is used to assess the probability that a patient has at least one node removed based on clinicopathologic factors; and (2) a negative binomial model (the count component of the model) compares the proportion of positive nodes among all removed nodes, for node-positive patients. To fit an adjusted model, a covariate was considered for the final model if the “univariate” *p* value from a likelihood ratio test with 2 degrees of freedom was <0.05. A manual backwards selection procedure was used to reduce the models. Bootstrapped confidence intervals on the parameter estimates were calculated based on 5,000 randomly bootstrapped samples. All tests were two-tailed with significance level *p* < 0.05 and computed using R software (v 2.15.2, 2012, www.r-project.org).

## Results

### Patient Population

A total of 84 women with invasive breast cancer who participated in the combined Phase III TcTM clinical trials and 115 women undergoing SLNB with TcSC at UCSD in the 12-month period after the conclusion of the second trial met the inclusion criteria and were injected with both radiotracer and VBD. Patient and tumor characteristics of the two groups are presented in Table 1. There were no significant differences in mean age or pathologic factors between groups, although the fTcSC cohort appeared to have slightly larger tumors.

**Table 1:** Clinicopathologic factors in clinically node-negative breast cancer patients undergoing SLN biopsy at a single institution

Clinicopathologic factor	[^99m^Tc]Tilmanocept (N = 84) n (%)	fTcSc (N = 115) n (%)	*P* value
Age	Mean 57.2 ± 10.8	Mean 59.7 ± 11.2	0.11
Tumor size			0.13
T1	61 (73)	74 (64)	
T2	22 (11)	33 (29)	
T3	1 (5)	8 (7)	
Tumor histology			0.47
IDC	48 (57)	72 (63)	
ILC	11 (13)	9 (8)	
Mixed (IDC + ILC)/other	25 (30)	34 (29)	
ER status			0.52
Positive	75 (89)	98 (85)	
Negative	9 (11)	17 (15)	
PR status			0.18
Positive	68 (81)	83 (72)	
Negative	16 (19)	32 (28)	
Her-2neu status			0.85
Amplified	14 (17)	18 (16)	
Unamplified	70 (83)	97 (84)	
Triple negative			0.22
Yes	5 (6)	14 (12)	
No	79 (94)	101 (88)	
Tumor grade (MBR)			0.25
1	32 (38)	42 (35)	
2	28 (33)	50 (43)	
3	24 (29)	23 (20)	
LVI			0.73
Present	17 (20)	26 (23)	
Absent	67 (80)	89(77)	
Surgery			0.33
Lumpectomy	66 (79)	83 (72)	
Mastectomy	18 (21)	32(28)	

*fTcSc* filtered**-**[^99m^Tc] sulfur colloid, *IDC* invasive ductal carcinoma, *ILC* invasive lobular carcinoma, *MBR* Modified-Bloom Richardson score, *LVI* lymphovascular invasion

### Intraoperative Node Identification and Pathology Findings

**Fig. 1:**
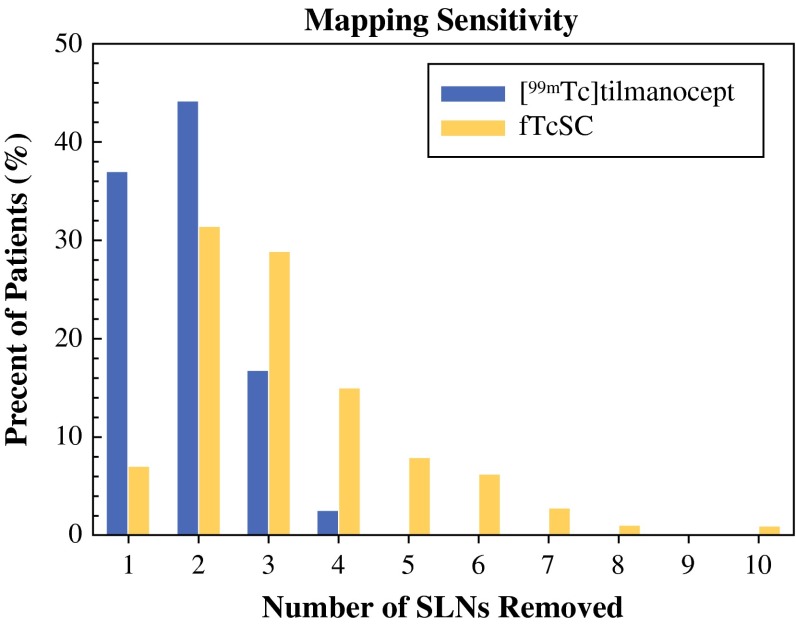
SLN mapping with [^99m^Tc]tilmanocept/VBD (*blue bars*) resulted in the removal of fewer total lymph nodes compared to fTcSC/VBD (*yellow bars*)

The intraoperative identification rate of axillary SLNs was 100 % for both groups. On average, the TcTM cohort had significantly fewer SLNs removed per patient compared with the fTcSC group [1.85 ± 0.78 (range 1–4) vs. 3.24 ± 1.62 (range 1–10), *p* < 0.001]. Figure 1 shows the distribution of the number of SLNs removed per patient according to radiotracer used (the mapping sensitivity). In the TcTM group, 96 % of patients had 3 or fewer nodes removed and no patient had more than 4 nodes removed. By comparison, nearly 20 % of patients in the TcSC cohort had more than 4 nodes removed. To investigate whether clinicopathologic factors might have accounted for differences in the number of nodes removed, a logistic regression model with outcome >3 SLNs removed confirmed that the fTcSC group was more likely to have more than 3 nodes removed even adjusting for these factors (*p* < 0.001). Apart from younger age, which appeared to be inversely associated with having a higher number of SLNs removed [odds ratio (OR) = 0.41 for age >60 vs. ≤60, 95 % CI = (0.19, 0.89)], no other factors were associated with number of nodes removed.

**Fig. 2:**
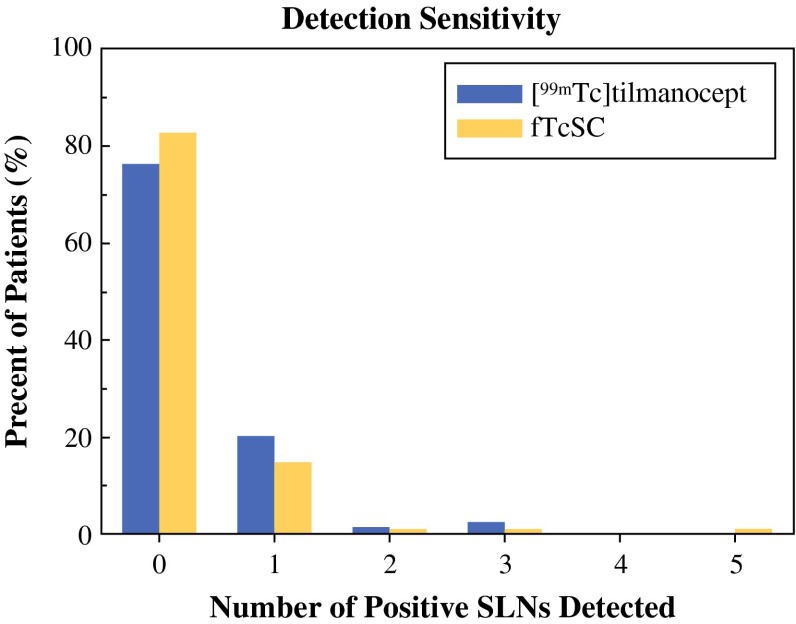
[^99m^Tc]tilmanocept (*blue bars*) and fTcSC (*yellow bars*) exhibited similar numbers of positive SLN detected, among those removed

Although there were fewer SLNs removed in the TcTM group, pathology analysis indicated that [^99m^Tc]tilmanocept and fTcSC exhibited similar sensitivity for detecting positive nodes (Fig. 2). The average number of positive nodes detected was 0.3 (95 % CI 0.16–0.43) for TcTM versus 0.23 (95 % CI 0.11–0.36) for fTcSC. There were slightly more patients identified with axillary metastasis in the TcTM cohort compared with the fTcSC group [20/84 (24 %, 95 % CI 0.15–0.33) of the patients vs. 20/115 (17 %, 95 % CI 0.11–0.25), *p* = 0.4]. The overall axillary metastasis rate among patients was 42 of 203 (20.7 %).

Among those with axillary metastasis (i.e., at least one detected positive node) in the TcTM group, a larger proportion of removed nodes were found to be positive [# positive nodes divided by # nodes removed for each patient: 0.73 (95 % CI 0.6–0.86, *n* = 20) for TcTM vs. 0.43 (95 % CI 0.32–0.54, *n* = 20) for fTcSC]. Thus, the rate of positive nodes among those removed was approximately 1.7 times greater (0.73/0.43 = 1.7) for TcTM compared with fTcSC.

To investigate the statistical significance of these observations after adjusting for clinical and pathological factors, we used a ZINB model, which models the probability of having positive node status using logistic regression, and then models the number of positive nodes among those patients who were node-positive using a negative binomial model, in a compound likelihood. This adjusted analysis confirmed a significantly higher rate of positive nodes among removed nodes for the patients mapped with TcTM compared with fTcSC (adjusted rate ratio = 5.52, 95 % CI 2.46, 12.39, *p* < 0.001). In addition, among all the covariates considered, the ZINB model showed that LVI status [odds for being node-negative for presence vs. absence = 0.03 (95 % CI, 0.0034–0.27)] and tumor size [odds for being node-negative for those with T2 or T3 vs. T1 = 0.11 (95 % CI, 0.018–0.61)] were independent predictors for having any positive SLN.

## Discussion

SLN biopsy is widely accepted in breast cancer surgery; however, false-negative rates in modern practice range up to 10 %; and the number of SLNs removed varies based on surgeon and tumor-related factors, with frequent removal of unnecessary nodes.^3^,^4^,^6^,^16^ Ideally, only the anatomically sentinel node(s) would be removed. Technetium 99m-tilmanocept (TcTM) is a receptor-targeted radiopharmaceutical that binds specificity to macrophages (CD-206 receptor) and was designed with the aim to achieve superior SLN-targeting during preoperative lymphoscintigraphy and intraoperative SLN identification.^8^,^17^ It remains to be determined whether improved targeting of these clinically relevant nodes can simultaneously improve biopsy accuracy and also limit the number of nodes removed.

In the current study, we found that on average significantly fewer SLNs were removed in patients mapped with TcTM + VBD compared with patients mapped with fTcSC + VBD (average number of nodes removed, 1.85 vs. 3.24, respectively). In the TcTM group, 96 % of patients had 3 or fewer nodes removed and no patients had more than 4 nodes removed. By comparison, nearly 20 % of patients in the TcSC cohort had more than 4 nodes removed. This rate is consistent with prior series evaluating the number of SLNs removed with various preparations of TcSC, where the reported ranges vary from 1–8 to 1–13 SLNs, and in 20 % of patients, more than 4 nodes are removed.^4^,^18^

Removal of fewer SLNs might potentially decrease patient morbidity. For example, the American College of Surgeons Oncology Group Z0010 trial (ACOSOG Z0010) found that a high number of SLNs removed (>5) was associated with increased incidence of seromas and infection.^19^ Additionally, removing fewer SLNs may lower the operative time and pathology cost of the procedure.^7^ However, removal of fewer nodes is not justified if diagnostic accuracy is not maintained. The minimal number of SLNs necessary for accurate axillary staging is heavily debated.^4^–^6^,^18^,^20^,^21^ Many authors have observed that removing a larger number of SLN(s) improves biopsy accuracy and that up to 4–5 nodes are needed in some patients to achieve acceptable false negative rates.^18^,^20^,^22^,^23^

It is important to consider that the removal of many nodes in a patient implies the removal of some higher echelon nodes.^1^,^24^–^26^ In order to study factors that may influence removal of nonsentinel nodes, we analyzed predictors of removal of more than 3 SLNs, using multivariate logistic regression. The threshold of 3 was based on studies evaluating lymphatic drainage of the breast, which demonstrate there are generally at most 3 parallel draining pathways from the breast.^24^–^26^ Therefore, removal of more than 3 SLNs implies identification of nodes that are anatomically nonsentinel. These nodes would not hold the same predictive capacity as first-draining nodes and thus can be clinically confusing when analyzed in the setting of SLN biopsy. The worst-case consequence of downstream node labeling is that a true anatomically sentinel node holding metastasis might be missed despite successful identification of “hot nodes” intraoperatively (which are truly higher echelon nodes). Thus, removal of a larger number of nodes alone would not necessarily improve biopsy accuracy, because it might lead to suboptimal targeting of first-draining nodes.

In our study, the decrease in number of nodes removed among patients mapped with TcTM did not come at the expense of a lower rate of detection of node-positive patients. A similar proportion of node-positive patients were observed in the TcTM cohort and the fTcSC cohort (24 vs. 17 %). This would be expected, as our populations were fairly homogenous in important clinicopathologic factors.

Importantly, despite removal of fewer total nodes by TcTM, the number of positive nodes removed per patient was very similar for the two radiotracers and was 0.3 for TcTM versus 0.26 for fTcSC. Considering only node-positive patients, the proportion of positive nodes among nodes removed was approximately 1.7 times greater (observed relative risk [RR] = 0.73/0.43 = 1.7) for TcTM compared with fTcSC. After adjusting for clinical and pathologic factors in a ZINB model, we found that TcTM remained significantly more likely to discover a positive node among the nodes removed. Because the model takes into account both the risk of node positivity and the proportion of positive nodes among the number of nodes removed, the clinical relevance is that fewer nodes were necessary to detect metastasis and that overall, nodes removed in the TcTM held greater predictive value. This would support the theory that there is improved targeting of the clinically relevant nodes in the TcTM group.

In our study, all patients were mapped with a dual-agent method (radiotracer + VBD), and we do not discriminate in our study between SLNs that were only hot versus only blue or both. However, because VBD was common to both radiotracer groups, it would follow to attribute technical differences to the radiolabeled agent. Additionally, many factors may influence the number of SLNs and although we controlled for some, many factors were not controlled for by nature of retrospective comparison (such as multiple radiologists performing injections, slight variance in technique). However, the most important determinant of number of SLNs removed at surgery is related to surgeon skill/experience, which is well controlled for in our study in that only two surgeons, each with more than 10 years of SLN surgery experience, performed all the surgeries.^1^ Finally, as aforementioned, it is impossible to evaluate the accuracy fully without performing a completion node dissection and calculating an FNR, which we did not do in our study. We provide compelling evidence for greater sensitivity for finding positive nodes with our ZINB mode; however, larger studies and evaluation of FNR In patients with cancers where SLN biopsy is not yet standard of care will more directly evaluate relative accuracy between agents. This is currently being done in a recently completed Phase III clinical trial evaluating the use of TcTM for SLNB in oral cavity squamous cell carcinoma.^27^ Technical outcomes of this trial were recently presented and contrasted to results from a prospective study of SLNB in SCC using TcSC. This study found TcTM provided improved overall accuracy (AC) and decreased FNR compared with TcSC (AC: 99 vs. 97 %, *p* = 0.02; FNR: 3 % vs. 10 %, *p* < 0.001, for TcTM and TcSC respectively).^28^ Thus, these results are consistent with the theory of maintained or improved staging accuracy with use of TcTM.

## Conclusions

We compared breast cancer patients undergoing SLN biopsy at a single institution as part of TcTM Phase III clinical trials to a similar cohort of patients undergoing SLN biopsy with fTcSC. We found that significantly fewer SLNs were removed on average in patients mapped with TcTM + VBD compared with those patients mapped with TcSC + VBD. However, TcTM + VBD identified a similar proportion of node-positive patients, and the number of positive nodes removed using the two radiotracers also was very similar. Our data suggest that a molecular-based mechanism of SLN identification may produce superior targeting of the true SLN(s) and thus reduce the total number of nodes necessary for accurate axillary staging in early stage breast cancer patients.
